# Clinical characteristics and identification of risk factors of testicular torsion in children: A retrospective study in a single institution

**DOI:** 10.3389/fsurg.2022.1040487

**Published:** 2023-01-06

**Authors:** Kaiping Zhang, Yin Zhang, Min Chao

**Affiliations:** Department of Urology, Anhui Provincial Children’s Hospital/Children’s Hospital of Fudan University (Affiliated Anhui Branch), Hefei, China

**Keywords:** orchiectomy, clinical characteristics, risk factors, testicular torsion, orchidopexy

## Abstract

**Background:**

Testicular torsion (TT) is a common urological emergency posing serious health problem in children. Prompt diagnosis and treatment of TT are very important for children to protect the affected testis. The aim of this study was to evaluate the historical features, physical examination findings, laboratory tests, and ultrasound examinations in children with TT, as well as to identify the predictors of testicular salvage in children.

**Materials and methods:**

We conducted a retrospective record of clinical findings, laboratory data, ultrasound findings, operating results, and the results of follow-up in hospitalized children with TT from November 2004 to December 2021. A multivariable logistic regression model was used to identify predictors of testicular salvage.

**Results:**

A total 102 hospitalized children who presented with TT were included. Patients were aged from 1 month to 16 years, with a median age of 7.71 years. TT is significantly more common in the winter. Of these patients, 77 torsions were left-sided, 24 were on the right side, and only 1 was on bilateral sides. Meanwhile, we detected that 88 children suffering from TT had intravaginal torsion of the spermatic cord, and the rest were outside. Anticlockwise torsion was found in 65 cases (63.73%) and clockwise torsion in 37 (36.27%). As a result, 60 underwent orchidectomy, while 42 had a scrotal exploration with fixation of the testis. Multivariate analysis showed that cause of TT, time to intervention, white blood cell (WBC), and mean platelet volume (MPV) were correlated with the risk of a surgical outcome.

**Conclusion:**

Only a small proportion of TT children received timely surgical management. Testicular salvage can be predicted by cause of TT, time to intervention, WBC, and MPV. Early scrotal exploration based on careful physical examination decreases the risk of misdiagnosis of spermatic cord torsion. A certain percentage of children with TT presenting with scrotal trauma or epididymo-orchitis should have their testicles checked to make sure that they do not have torsion, especially those who visit in the cold season.

## Introduction

Testicular torsion (TT) is one of the most common diseases encountered in a pediatric hospital emergency department, with its incidence rate being about 1 in 4,000 males under the age of 25 (1). It is often caused by obstruction of testicular blood flow through a twisting of the longitudinal axis of the spermatic cord. TT is a time-dependent urgent event (2). Active management can relieve the suffering of patients, protect the testicular function, and improve male fertility (3, 4). It is well known that the two main determinants for testicular salvage include the degree of cord twisting and the timing of patient presentation after symptom onset. Thus, prompt diagnosis and treatment of TT should be performed immediately as long-term torsion may result in pain, testicular atrophy, or even totally loss of the affected testis.

Children who are diagnosed with TT usually have a loss of the cremasteric reflex on the affected side and abnormal testicle direction. The most common sign and symptom of torsion is sudden onset, severe, and unrelenting scrotal or/and inguinal pain (5). Some children may suffer from fever, nausea, and vomiting (6). However, it is often difficult to distinguish TT from other causes of acute pediatric scrotum syndrome such as epididymo-orchitis, infected hydrocele, and torsion of the appendix of testis. Up to 15% of children presenting with acute scrotum syndrome are diagnosed with torsion (7). Even an experienced pediatrician cannot make a clear distinction between TT and epididymo-orchitis with full assurance. Thus, physical examination findings are important for the diagnosis of TT. In addition, color Doppler ultrasonography of the testis is widely used for fast examination in the diagnosis of TT. In general, the torsed testicle may be irreversibly lost if the symptom duration remains over 6 h after initial pain (8). Delayed surgical management of TT is often made owing to lack of experience on the identification and diagnosis of TT.

The aim of this retrospective study is to summarize the clinical manifestations, physical examinations, auxiliary examinations, and surgical outcomes of TT in order to improve the diagnosis and treatment, as well as to explore the risk factors for predicting the outcomes of pediatric patients diagnosed with TT at the children's hospital in China from November 2004 to December 2021.

## Methods

Consent was not required from the Ethics Committee because of the retrospective nature of this study. Written informed consent was routinely obtained from each surgical patient or the legal guardian/next of kin for minors.

A retrospective analysis of the medical records on TT from November 2004 to December 2021 was conducted at our institution. Only cases confirmed by scrotal exploration were consecutively included in this study. Patients whose parents refused surgical exploration were excluded from this study in addition to negative scrotal exploration, neonatal torsion, or testicular appendix torsion. We extracted clinical data including age, duration of symptoms onset, birth weight, gestational age of mother, mode of delivery, feeding pattern, concomitant diagnose, ultrasound characteristics, laboratory testing, and direction and degree of spermatic cord twisting. Meanwhile, some children also had other concomitant diagnoses, namely, contained hydrocele, cryptorchidism, inguinal hernia, or testicular microlithiasis. Children diagnosed with TT underwent orchiectomy and/or surgical detorsion. The contralateral testis was routinely fixed in the same fashion unless parents refused to testicular fixation.

The patients were followed up at 1, 3, and 6 months postoperatively, and then examined once a year in the clinic. In addition, color Doppler ultrasound was widely used as a helpful tool for follow-up visits. We collected the number of testicular atrophies based on ultrasound after orchiopexy (defined as a >20% difference in volume compared to the contralateral testis).

### Surgical management

First, the scrotal skin and tunica vaginalis of the affected side were incised under general anesthesia. Second, a small incision was made in the tunica albuginea to check the blood supply, and then the affected testis was covered with a piece of moistened gauze with warm saline for 10–30 min. Third, after a moment, the affected testis was rechecked for potential salvageability. Generally, orchidopexy was performed if the testicular blood flow was restored. Otherwise, the nonviable testis was removed, and then fixation of contralateral testis was performed. In practice, an in-depth communication with parents was made to decide which kind of surgery (orchidopexy or orchiectomy) is suitable for their children in the face of coagulative necrosis of the testicular parenchyma. In Meanwhile, parents also decided whether to fix the contralateral testis.

### Statistical analysis

SPSS version 19.0 (SPSS Inc., Chicago, IL, United States) was applied for statistical analysis. Categorical variables were presented as ratios (%) and continuous variables were presented by mean ± standard deviation (SD). All continuous variables were tested for normality using the Shapiro–Wilk test. For normal distributed data, Student's *t* test was used. For non-normal data distribution, the Kruskal–Wallis test was applied instead. Comparisons were conducted by *χ*^2^ test or Fisher's exact test for dichotomous outcomes and unordered multiple outcomes, and by Kruskal–Wallis test for ordered multiple outcomes. A *P*-value less than 0.05 was considered statistically significant.

## Results

A retrospective record of 102 patients who were diagnosed with TT to our institution was made after surgery from November 2004 to December 2021. The primary characteristics of the patients are presented in Table 1. The age of the patients enrolled ranged from 1 month to 16 years, wherein TT mainly occurred in the first year of life and between the ages of 13 and 16 years. Most patients had a history of breast feeding and normal birth weight. The duration of pain or symptoms was 1 h to 15 days. The common causes of TT included scrotal trauma and epididymo-orchitis. However, most causes were still unknown. Thirty patients had concomitant diagnosis, namely, cryptorchidism, hydrocele, inguinal hernia, and testicular microlithiasis. Acute scrotal pain was the most common finding in the TT patients, followed by lower abdominal or inguinal pain. Moreover, we also found that TT often occurs in winter compared to other seasons (Table 1).

**Table 1:** Details of patients with TT.

Variables	Data, *n* (%)
Age	
Median age (range), years	7.71 (0.08–16)
Feeding pattern	
Breast feeding	64 (62.75)
Artificial feeding	18 (17.65)
Mixed feeding	20 (19.60)
Duration of symptoms (h)	
<12	24 (23.53)
12–24	27 (26.47)
>24	51 (50.00)
Cause of TT	
Trauma	7 (6.86)
Epididymo-orchitis	9 (8.82)
Unknown	86 (84.32)
Birth weight (g)	
Low (<2,500)	6 (5.88)
Normal (2,500–4,000)	95 (93.14)
High (>4,000)	1 (0.98)
Concomitant extrascrotal symptoms	
None	93 (91.18)
Abdominal pain	2 (1.96)
Nausea and/or vomit	1 (0.98)
Fever	1 (0.98)
Inguinal pain/mass	5 (4.90)
Other diagnosis	
Cryptorchidism	12 (11.77)
Hydrocele	11 (10.78)
Inguinal hernia	5 (4.90)
Testicular microlithiasis	2 (1.96)
None	72 (70.59)
Season	
Spring (March to May)	15 (14.71)
Summer (June to August)	26 (25.49)
Autumn (September to November)	24 (23.53)
Winter (December to February)	37 (36.27)

TT, testicular torsion.

These boys underwent surgery promptly after hospitalization. The common complications included bleeding and scrotal edema after operation. No serious complication was detected. Operative findings and surgical outcomes in patients with TT are shown in Table 2. From Table 2, the range of twisting in the torsion could be identified as 90°–1,080°. Of these patients, 77 (75.49%) torsions were left-sided, 24 were on the right side, and only 1 was bilateral sides. Meanwhile, we detected that 88 children suffering from TT had intravaginal torsion of the spermatic cord, and the rest were outside. Anticlockwise torsion was found in 65 cases (63.73%) and clockwise torsion in 37 (36.27%). As a result, 60 underwent orchidectomy, while 42 had a scrotal exploration with fixation of the testis. In addition, the contralateral testis was fixed in 33 of 102 (32.35%) cases according to the agreement from their guardians. Of the cases of testicular salvage, two patients developed subsequent testicular atrophy after follow-up of half a year.

**Table 2:** Operative findings and surgical outcomes in patients with TT.

Variables	Patients with TT, *n* (%)
Torsion degree	
0°–180°	9 (8.82)
181°–360°	39 (38.24)
361°–540°	13 (12.74)
>540°	41 (40.20)
Laterality	
Right side	24 (23.53)
Left side	77 (75.49)
Both sides	1 (0.98)
Torsion direction	
Clockwise	37 (36.27)
Anticlockwise	65 (63.73)
Position to cavity of tunica vaginalis	
Inside	88 (86.27)
Outside	14 (13.73)
Outcome	
Orchidopexy	42 (41.18)
Orchiectomy	60 (58.82)
Fixation of contralateral testis	
Yes	33 (32.35)
No	69 (67.65)

TT, testicular torsion.

To determine the factors influencing the surgical procedures, patients were classified into two groups depending on whether they underwent orchidopexy or orchiectomy. On univariable analysis, we found that those in the orchidopexy group tended to have significantly lower degrees of twisting (*P* = 0.002) and shorter duration of symptoms (*P* = 0.001) when compared to their viable counterparts (Table 3). In addition, there was a statistical difference in the group of TT causes (*P* = 0.003). There was no statistical difference in other variables between the two groups (*P* > 0.05).

**Table 3:** Comparison between patients underwent orchidopexy and orchiectomy.

Variable	Orchidopexy (*n* = 42)	Orchiectomy (*n* = 60)	*P-*value
Age (years; mean ± SD)	8.32 ± 4.87	6.89 ± 5.50	0.177
Degree of torsion (°; mean ± SD)	437.14 ± 202.30	571.50 ± 217.84	0.002
Duration of symptoms (h; mean ± SD)	31.64 ± 36.54	82.42 ± 88.09	0.001
Cause of TT			0.003
Trauma, *n* (%)	4 (9.52)	3 (5.00)	
Epididymo-orchitis, *n* (%)	8 (19.05)	1 (1.67)	
Unknown, *n* (%)	30 (1.43)	56 (93.33)	
Season			0.506
Spring, *n* (%)	6 (14.29)	9 (15.00)	
Summer, *n* (%)	13 (30.95)	13 (21.67)	
Autumn, *n* (%)	7 (16.67)	17 (28.33)	
Winter, *n* (%)	16 (38.09)	21 (35.00)	
Laterality			0.616
Right side, *n* (%)	9 (21.43)	15 (25.00)	
Left side, *n* (%)	32 (76.19)	45 (75.00)	
Both sides	1 (2.38)	0 (0.00)	
Torsion direction			0.835
Clockwise, *n* (%)	16 (38.10)	21 (35.00)	
Anticlockwise, *n* (%)	26 (61.90)	39 (65.00)	
Position to cavity of tunica vaginalis			0.388
Inside, *n* (%)	38 (90.48)	50 (83.33)	
Outside, *n* (%)	4 (9.52)	10 (16.67)	

TT, testicular torsion; SD, standard deviation.

There were significant differences between orchidopexy and orchiectomy in terms of white blood cell (WBC) and mean platelet volume (MPV). However, there was no statistically significant difference in other hematologic parameters between the two groups (Table 4). Likewise, a multivariate analysis of the two groups revealed that cause of TT, time to intervention, WBC, and MPV were correlated with the risk of a nonsalvageable testis (Table 5).

**Table 4:** Hematologic parameters of the study groups.

Variable	Orchidopexy (*n* = 42)	Orchiectomy (*n* = 60)	*P-*value
WBC (10^9^/L, mean ± SD)	11.07 ± 3.09	12.32 ± 3.03	0.044
RBC (10^12^/L, mean ± SD)	4.78 ± 0.44	4.65 ± 0.37	0.108
Hb (g/L, mean ± SD)	132.48 ± 12.83	126.85 ± 15.73	0.058
Plt (10^9^/L, mean ± SD)	287.79 ± 85.32	313.03 ± 102.77	0.194
CRP (mg/L, mean ± SD)	8.60 ± 15.57	8.89 ± 11.78	0.916
PDW (fl, mean ± SD)	13.25 ± 2.63	13.45 ± 3.14	0.742
MPV (fl, mean ± SD)	10.07 ± 1.20	10.63 ± 1.38	0.035
HCT (L/L, mean ± SD)	37.45 ± 10.82	39.27 ± 5.45	0.266
MCV (fl, mean ± SD)	84.43 ± 4.26	86.88 ± 10.44	0.106

WBC, white blood cell; RBC, red blood cell; MPV, mean platelet volume; CRP, C-reactive protein; PDW, platelet distribution width; HCT, hematocrit; MCV, mean corpuscular volume; SD, standard deviation.

**Table 5:** Results of multivariable analysis between patients underwent orchidopexy and orchiectomy by logistic regression analysis.

Variable	OR (95% CI)	*P-*value
Cause of TT	5.120 (1.752–14.960)	0.003
Torsion degree	1.002 (1.000–1.005)	0.080
Duration of symptoms	1.025 (1.010–1.041)	0.001
WBC	1.259 (1.048–1.511)	0.014
MPV	1.959 (1.211–3.168)	0.006

TT, testicular torsion; WBC, white blood cell; MPV, mean platelet volume; OR, odd ratio; CI, confidence interval.

## Discussion

TT is a surgical emergency and may affect testicular function and male fertility if not treated timely. Approximately 30% children with acute scrotum had delayed surgical management of TT because they were not first treated in a tertiary hospital. Patients transferred from primary and secondary healthcare units were more likely to delay management owing to the lack of experience on the identification and diagnosis of TT. Thus, it is important to continuously promote public awareness of TT, and improve pediatrician ability of diagnosis and therapy of TT from primary and secondary healthcare units.

Acute severe scrotal pain is a symptom that can be identified in clinical presentation. Other common signs and symptoms of torsion, as well as other causes of acute pediatric scrotum syndrome, include sudden abdominal or inguinal pain, nausea, and vomiting. Not all TT cases have acute severe scrotal pain. Actually, we often need to make an accurate differential diagnosis of an undescended TT from acute abdomen or incarcerated hernia through proper abdominal and inguinal examination, especially for defective children or infants. It may be difficult to distinguish from other causes of acute pediatric scrotum syndrome such as epididymo-orchitis, infected hydrocele, and torsion of the appendix of testis. Surgery is the primary treatment after suspicion of TT. The duration of pain or symptom was a critical factor of surgical selection (9). However, the definition of delayed surgery was still not well established. To date, the “golden window of opportunity” to testicular salvage after symptom onset is suggested to be 0–6 h, as not intervening within this time decreases function and increases the rate of orchiectomy (10, 11). If treated within 6 h from the onset of symptoms, 90%–100% affected testicles will be saved. If symptoms last beyond 12 h, salvage rates will decrease to 50%. The incidence of testicular salvage is even less than 10% over 24 h (12).

Previous studies have always have explored the risk factors of TT and found that age is an important risk factor. TT can occur at any age but usually occurs in young males, especially in the first year of life and around puberty (13, 14). Children under the age of 6 could not express themselves and judge the severity of the scrotal pain, resulting in delayed management of TT (15). There was a similar age distribution in our study, with peaks of incidence during age less than 1 (18.63%, 19/102) and around puberty (39.22%, 40/102). In addition, environmental temperature has been associated with variations in the incidence of TT. Cremaster muscle spasm in response to cold weather has been implied in the occurrence of TT. Cold weather, as well as the resultant cremasteric hyperactivity, can be risk factors for torsion in individuals with unfavorable anatomy including testicles with a greater horizontal axis (16). It has been reported that over 75% of TT cases were found more commonly in cold season with a low ambient temperature below 15°C (17). Furthermore, Ekici et al. (18) reported that approximately 46% of TT cases were detected in the winter months in their study. This is similar to the rate found in our present study. More TT cases were detected during winter (36.25%) in the present study. Scrotal trauma also may result in TT by an abrupt vigorous contraction of the cremaster muscle, accounting for 4%–8% of all TT cases (19, 20). Traumatic torsion was often observed in patients with risk factors for TT such as bell clapper deformity and horizontal lie of the testes (19). These patients required a high index of suspicion of TT, and then an urgent ultrasound scan was performed. Moreover, about 5% of patients who presented with torsion suffered from cryptorchidism, which may be explained by incorrect spasm or contraction of cremasteric muscle (21, 22).

Debate always exists regarding contralateral testicle fixation, arguing that the low risk of contralateral torsion outweighs the risk of complications from orchidopexy (23). The exact risk of contralateral torsion is still unknown. However, a previous study found that the contralateral testis often had some predisposing factors in 78% of pubertal TT cases, such as the bell clapper deformity and horizontal lie of the testes (24). Furthermore, some bilateral torsion may present with unilateral signs (25). In our study, there was only one contralateral torsion 1 year after orchiectomy. Given these findings, the contralateral testicle should undergo fixation to reduce the risk of torsion.

Though we collected the medical records of TT cases from the past decade, some limitations still existed. First, our study design was a retrospective study and restricted the accuracy of results. Second, only patients with TT in our pediatric center were included, which could not completely represent the general condition. Finally, some potential or undiscovered factors may affect the ultimate results, such as family history of TT, medical insurance, and guardians’ work. Therefore, a prospective, well-designed, and large-scale study should be performed.

## Conclusion

Only a small proportion of TT children received timely surgical management. Testicular salvage can be predicted by cause of TT, time to intervention, WBC, and MPV. Early scrotal exploration based on careful physical examination decreases the risk of misdiagnosis of spermatic cord torsion. A certain percentage of children with TT presenting with scrotal trauma or epididymo-orchitis should have their testicles checked to make sure that they do not have torsion, especially those who visit the in cold season.

## Data Availability

The original contributions presented in the study are included in the article, further inquiries can be directed to the corresponding author.
